# The effects of a self-learning package on mothers' knowledge and practices towards caring for their children with phenylketonuria

**DOI:** 10.25122/jml-2022-0258

**Published:** 2023-02

**Authors:** Amira Khalil, Eman Amin, Safy Salah Eldin Alrafay, Ola Ali Khalifa

**Affiliations:** 1 Pediatric Nursing Department, Faculty of Nursing, Ain Shams University, Cairo, Egypt; 2 Medical Genetics Department, Faculty of Medicine, Ain Shams University, Cairo, Egypt

**Keywords:** phenylketonuria, mothers, practice, self-learning package

## Abstract

The objective of this study was to evaluate the effect of a self-learning package on mothers' knowledge and practices towards caring for their children with phenylketonuria. A pre/post quasi-experimental study was conducted, including 128 mothers of children diagnosed with phenylketonuria. A specifically designed and validated questionnaire was used to evaluate mothers' knowledge and reported practices toward their children before and after participating in the educational program. There was a highly positive association between knowledge and reported practice (.674 and .398). The self-learning package had a positive impact on mothers' knowledge and practices. Consequently, educational programs should be provided to all mothers of newly diagnosed cases to improve their children's adherence to the therapeutic regimen.

## INTRODUCTION

Phenylketonuria (PKU) is a rare genetic disorder that results from an inborn error in phenylalanine metabolism. PKU is inherited in an autosomal recessive manner, meaning that an affected person inherits the specific gene mutation from both healthy parents who are carriers. A double dose of the mutated gene in the affected person results in a deficiency of phenylalanine hydroxylase, which is responsible for breaking down phenylalanine (PHE) into tyrosine (TYR). If left untreated, serum PHE levels become more elevated and harm the developing central nervous system of the affected person [1].

Phenylketonuria symptoms can range from mild to severe depending on different levels of phenylalanine hydroxylase deficiency. Classical PKU is the most common and severe form of PKU with little or no PAH enzyme activity [2]. The incidence of PKU varies significantly between countries. For example, 1 in 10,000 newborns in the United States is impacted by this condition. With 1 in 2,600 births, Turkey has the highest reported rate in the world, whereas Finland and Japan have incredibly low rates, with fewer than one PKU case per 100,000 births [3]. The incidence of PKU in Egypt is unknown. However, the total number of children with this disorder at the metabolic clinic, Department of Medical Genetics, Faculty of Medicine, Ain Shams University, was around 730 cases.

Nursing interventions for children with PKU are pivotal. In particular, nurses should inform caregivers about strict adherence to dietary protocol. Children suffering from the undesirable effect of illness require continuing support and nursing care, which is the mainstay of such patients [4]. Mothers play a pivotal role in the management of childhood PKU which needs a diverse range of complex skills and learning programs to deal with the children's disabilities and to maintain a special rehabilitation and diet program [5, 6]. A self-learning package (SLP) is a document containing all educational objectives necessary for a learner to attain learning objectives independently of the teacher. The learner can take over a large part of the training while the teacher remains available [7].

PKU is a potentially serious inherited disorder, and without early diagnosis and correct treatment, most PKU children will develop irreversible brain damage, neurological problems, and behavioral abnormalities. Lack of awareness about the Newborn Screening (NBS) program and the best methods to care for children with PKU contributes to adverse outcomes. Therefore, this study aimed to evaluate the effect of a self-learning package on mothers' knowledge and practices towards caring for their children with PKU.

## MATERIAL AND METHODS

### Research design

This study used a pre/post quasi-experimental research design to achieve the aim of the study. We used a purposely designed and validated instrument to evaluate mothers' knowledge and reported practices before and after the self-learning package. The study was conducted at the Metabolic Clinic, Department of Medical Genetics, Faculty of Medicine, Ain Shams University. The study was conducted between May 1^st^, 2021, and January 31^st^, 2022. During this time, the researcher was available in the study setting for 4 hours 2 days/a week (Monday and Wednesday) according to the clinic time. A sample size of at least 128 mothers having children with PKU was satisfactory for detecting an effect size of at least 0.25 (small to moderate effect size) using paired t-test at a significance level of 0.05 and a power of 0.80. This sample size was satisfactory to estimate the proportion of participants who improved knowledge/reported practice of 50% with a 95% confidence interval of 20% (40% to 60%).

This study hypothesized that implementing a self-learning package would positively affect the knowledge and practices of mothers towards caring for their children with PKU.

### Research instruments

After an extensive review of the literature, the researcher developed an Arabic questionnaire consisting of three parts:

Part I: Socio-demographics: including age, level of education, residence, and income.

Part II: Mothers' knowledge about PKU adapted from van Spronsen *et al*. [8]. This part included questions regarding the definition, causes, symptoms and signs, diagnosis, prevention of potential complications, and different treatment methods (dietary restriction, special medical formula, and medical treatment). The response to each of the 6 items consists of three options: incorrect/don't know (0) and correct (1). The total knowledge score was categorized as satisfactory (≥60.0%) or unsatisfactory (<60.0%).

Part III: Mothers' reported practice was assessed after reviewing the current literature [9] divided into 8 domains: (1) medical diet (7 items), (2) follow-up of baby weight at home (5 items), (3) follow up of child weight at home (5 items), (4) follow up of length at home, (5) follow up of height at home (10 items), (6) follow up of BMI (4 items), (7) dental care (7 items), and (8) physical activity (5 items). Response to each item consisted of two options: no (0) and yes (1). The total practice score was categorized as good reported practice (score ≥70%), average reported practice (50–70%), and poor reported practice (<50%).

### Pilot study

A pilot study was conducted on a group of 13 mothers (10%) prior to official data collection to assess the feasibility, duration, and cost of a full-scale research project. As no modifications were made to the study, the participants in the pilot were included in the main study as well.

### Study framework

The study was conducted in the following phases: assessment, planning, implementation, and evaluation. In the assessment phase, a pre-implementation assessment of the mothers' knowledge and reported practices of PKU was conducted through interviews using the questionnaire described above. In the planning phase, the goals, priorities, and expected outcomes from the assessment phase were formulated to meet mothers' needs and create a self-learning package. The researcher verified sources (such as books/websites) for mothers to conduct a self-learning package, and the researchers were available for any questions or feedback from mothers. During the implementation phase, mothers attended two consecutive educational sessions, with each session ranging from 30 to 40 min. The first session focused on knowledge, including the definition of PKU, causes, signs and symptoms, diagnosis, prevention of potential complications, and different treatment methods. The second session was related to mothers' practices, including formula preparations, follow-up for growth (weight, height or length, BMI), dental care, and physical activity. In each session, small groups (ten mothers each) were led through small-group discussions and assignments directed by the researcher. Finally, in the evaluation phase (post-test), the same pretest questionnaire was used to assess the effect of the self-learning package on mothers' knowledge and reported practices towards caring for their children with phenylketonuria. The questionnaire was administered 1 month after the end of the training program.

### Statistical analysis

Data were coded and entered into IBM SPSS Statistics for Windows, version 24 (IBM Corp., Armonk, NY, USA) and reviewed to detect any entry errors. Qualitative data was presented as a number and a percent, and quantitative data were described as mean or standard deviation. Student's t-test was used to determine differences between the means of the two groups. Qualitative categorical variables were compared using the Chi-square test. Spearman rank correlation was used to assess the inter-relationships between quantitative and ranked variables. The results were considered statistically significant at P≤0.05. The tools developed for this study were evaluated using Cronbach's alpha coefficient test. The internal consistency reliability (Cronbach's α) for the predesigned questionnaire was good for knowledge (Cronbach's α=0.799) and good for practice (Cronbach's α=0.813).

Of the 128 surveyed mothers, the majority (55.5%) were aged 20 to 30, with an average age of 23.7 years (±8.2). Almost two-thirds (67.2%) identified as housewives, while 38.2% had intermediate levels of education. In addition, a high proportion of participants (64.1%) reported insufficient income and lived in rural areas (Table 1).

**Table 1:** Demographic characteristics of participants (n=128).

Items	N	%
**Age**		
<20	21	16.3
20 to <30	71	16.3
30 to <40	24	18.8
≥40	12	9.4
**Mean±SD=23.7±8.2**		
**Educational level**		
Basic education	46	36
Intermediate education	49	38.2
Higher education	18	14.1
Postgraduate	15	11.7
**Work status**		
Working	42	32.8
Not working/housewife	86	**67.2**
**Monthly Income**		
Insufficient	82	**64.1**
Sufficient	46	35.9
**Residence**		
Rural	80	62.5
Urban	48	37.5

Before the self-learning package, 75.8% of the participants had inadequate knowledge about PKU. However, this number significantly reduced to 48.4% after completing the training. In addition, there was a high statistically significant difference in the total knowledge scores before and after participating in the training (p-value=0.000), as shown in Table 2.

**Table 2:** Mothers' knowledge regarding PKU pre/post-self-learning intervention (n=128).

	Pre		Post		χ^2^	P-value
	N	%	N	%		
**Satisfactory knowledge**	31	24.2	66	51.6	11.580	.000**
**Unsatisfactory knowledge**	97	75.8	62	48.4	-	.000**
**Total mean score Mean±SD**	**6.11±4.33**		**14.81±1.68**		**t-test 10.523**	.000**

** – Highly significant.

We found a significant improvement in self-reported practices after educational training. More mothers reported monitoring medical diets (57%) and tracking the baby's length (92.9%) after the training than before (37.5% and 42.8%, respectively). Monitoring body mass index also improved, with 46.8% of mothers reporting good practice after the training compared to just 1.5% before. Similarly, more mothers reported good dental care practices (76.6%) and physical activity (60.1%) after the training, compared to just 5.4% and 21.9% before, respectively (Table 3).

**Table 3:** Mothers' reported practices regarding PKU pre/post-self-learning package (n=128).

Items		Pre (n=128)		Post (n=128)		χ^2^	P-value
		No	%	No	%		
**1. Measurement of medical diet**	Poor	70	54.7	13	10.2	15.098	<0.01**
	Average	48	**37.5**	73	**57**		
	Good	10	7.8	42	32.8		
**2. Follow up on weight** 2A. Baby weight (n=42)	Poor	0	0	0	0	17.008	<0.01**
	Average	9	21.4	2	4.8		
	Good	33	78.6	40	95.2		
2B. Child weight (n=86)	Poor	10	11.6	3	3.5	14.760	<0.01**
	Average	31	24.2	16	18.6		
	Good	45	35.2	67	77.9		
**3. Follow-up on height** 3A. Baby length (n=42)	Poor	12	28.6	0	0	16.003	<0.01**
	Average	12	28.6	3	7.1		
	Good	18	**42.8**	39	**92.9**		
3B. Child height (n=86)	Poor	32	37.2	2	2.3	15.002	<0.01**
	Average	24	27.9	13	15.1		
	Good	30	34.9	71	82.6		
**4. Follow-up on body mass index**	Poor	88	68.8	18	14.1	13.661	<0.01**
	Average	38	29.7	50	39.1		
	Good	2	**1.5**	60	**46.8**		
**5. Dental care**	Poor	49	38.3	4	3.1	16.021	<0.01**
	Average	72	56.3	26	20.3		
	Good	7	**5.4**	98	**76.6**		
**6. Physical activity**	Poor	48	37.5	13	10.2	18.055	<0.01**
	Average	52	40.6	38	29.7		
	Good	28	**21.9**	77	**60.1**		

**Figure 1:**
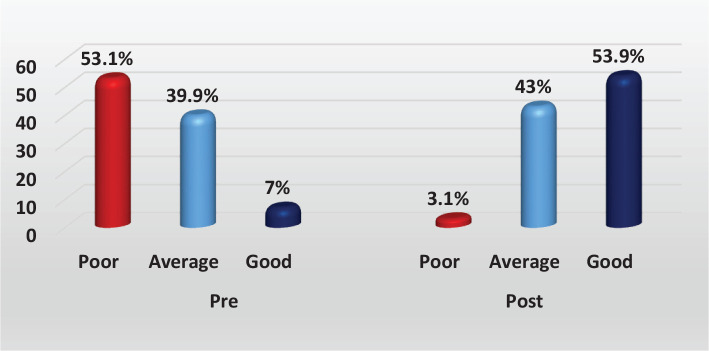
Distribution of total self-reported practice pre–post self-learning package (n=128).

Figure 1 reveals that more than half of the participants had poor practice levels before SLP, while only 3.1% had poor practice levels after SLP. There was a high statistically significant difference in the total level of practice among participants at different time points.

## DISCUSSION

The researcher used the self-learning package (SLP) to design instructional activities that guide the learner in independently achieving learning objectives. Improving the cognitive and psychomotor domains by acquiring information and applying it into practice facilities self-evaluation and makes mass teaching possible with high efficiency and high availability [10].

The majority of participants were in the age range of 20 to 30 years old, with an average age of 23.7 years and a standard deviation of 8.2 years. This age range is consistent with the typical childbearing age. This finding agrees with Mortazavi *et al*. [11], who showed that more than half of the participants were between 18 and 35 years old. Conversely, this finding disagrees with Prasetyo *et al*. [12], who had a mean±SD age of 31.23±5.72.

In addition, more than two-thirds of women did not work, and less than two-thirds had insufficient income. This result might be explained by the fact that most women quit work to provide care for their children, especially if their children have chronic illnesses. This result agrees with Etemad *et al*. [13], who revealed that nearly two-thirds of their mothers were not workers and had insufficient income. Furthermore, less than two-thirds of the participants were from rural areas, which disagrees with Witalis *et al*. [14], who found that more than two-thirds of the mothers were from urban areas. The level of knowledge regarding phenylketonuria significantly increased following the educational intervention, which is supported by Abd-Elkodoos *et al*. [15] and EL-Ghadban *et al*. [16], who mentioned that less than one-third of caregivers had good knowledge levels pre-self-learning module. However, after the self-learning module, more than half (52.5%) had a good knowledge level. On the other hand, Gurbuz *et al*. [17] found that most participants had good knowledge scores before the PKU program. When mothers' knowledge of PKU was compared, there was no significant difference before (7.40±1.62) and after (7.72±1.80) the program.

Our study also showed that less than one-third of the participants reported good practice regarding follow-up measurements of medical diet after SLP, while only 7.8% reported good practice before SLP. This result might be due to parents' dietary adherence struggles with their children from school age onwards. This result contrasts Ford *et al*. [18], who reported that 89% of the mothers had good practice regarding follow-up of measurement of medical diet after the educational program.

Less than half of mothers reported good practice regarding follow-up of BMI post-SLP, while only 1.5% reported good practice pre-SLP. Additionally, there was a high statistically significant difference regarding self-reported mothers' practice pre- and post-SLP. This finding might be due to the effectiveness of the self-learning package in improving mothers' practice, which agrees with Zaky *et al*. [19]. Furthermore, dental care practice was also improved after the educational program, corresponding to the findings of George *et al*. [20].

More than half of the mothers had poor practice levels before SLP, which considerably decreased after the intervention (3.1%). Additionally, there was a highly significant difference in the total level of practice before and after the intervention, corresponding with Rahgoi *et al*. [21].

## CONCLUSIONS

The results of this study showed that prior to the self-learning program (SLP), over half of the participants had inadequate practices and knowledge levels, whereas post-SLP, more than half demonstrated improved practices and knowledge. It is recommended that self-learning resources be made available to all mothers of children with PKU, both new and old cases, to enhance their adherence to the therapeutic regimen. Further research with larger sample sizes and in various locations in Egypt, with a focus on mothers of children with PKU, is necessary to improve their practices and enhance the quality of life of their children. The healthcare team should also prioritize the support of these mothers.
